# *Panax notoginseng* saponins inhibits NLRP3 inflammasome-mediated pyroptosis by downregulating lncRNA-ANRIL in cardiorenal syndrome type 4

**DOI:** 10.1186/s13020-023-00756-2

**Published:** 2023-05-08

**Authors:** Ying Xu, Luxi Cao, Wenli Zou, Rizhen Yu, Wei Shen

**Affiliations:** 1 Urology & Nephrology Center, Department of Urology, Zhejiang Provincial People‘s Hospital, Affiliated People‘s Hospital, Hangzhou Medical College, Hangzhou, 310014 Zhejiang China; 2 Urology & Nephrology Center, Department of Nephrology, Zhejiang Provincial People‘s Hospital, Affiliated People‘s Hospital, Hangzhou Medical College, No. 158, Shangtang Road, Hangzhou, 310014 Zhejiang China

**Keywords:** *Panax notoginseng* saponin, Cardiorenal syndrome type 4, LncRNA-ANRIL, Pyroptosis, Cardiac function

## Abstract

**Objective:**

Cardiorenal syndrome type 4 (CRS4) is a complication of chronic kidney disease. *Panax notoginseng* saponins (PNS) have been confirmed to be efficient in cardiovascular diseases. Our study aimed to explore the therapeutic role and mechanism of PNS in CRS4.

**Methods:**

CRS4 model rats and hypoxia-induced cardiomyocytes were treated with PNS, with and without pyroptosis inhibitor VX765 and ANRIL overexpression plasmids. Cardiac function and cardiorenal function biomarkers levels were measured by echocardiography and ELISA, respectively. Cardiac fibrosis was detected by Masson staining. Cell viability was determined by cell counting kit-8 and flow cytometry. Expression of fibrosis-related genes (COL-I, COL-III, TGF-β, α-SMA) and ANRIL was examined using RT-qPCR. Pyroptosis-related protein levels of NLRP3, ASC, IL-1β, TGF-β1, GSDMD-N, and caspase-1 were measured by western blotting or immunofluorescence staining.

**Results:**

PNS improved cardiac function, and inhibited cardiac fibrosis and pyroptosis in a dose-dependent manner in model rats and injured H9c2 cells (*p* < 0.01). The expression of fibrosis-related genes (COL-I, COL-III, TGF-β, α-SMA) and pyroptosis-related proteins (NLRP3, ASC, IL-1β, TGF-β1, GSDMD-N, and caspase-1) was inhibited by PNS in injured cardiac tissues and cells (*p* < 0.01). Additionally, ANRIL was upregulated in model rats and injured cells, but PNS reduced its expression in a dose-dependent manner (*p* < 0.05). Additionally, the inhibitory effect of PNS on pyroptosis in injured H9c2 cells was enhanced by VX765 and reversed by ANRIL overexpression, respectively (*p* < 0.05).

**Conclusion:**

PNS inhibits pyroptosis by downregulating lncRNA-ANRIL in CRS4.

**Supplementary Information:**

The online version contains supplementary material available at 10.1186/s13020-023-00756-2.

## Highlights

1. PNS improved cardiac function and suppressed pyroptosis in CRS4 rats.
2. PNS inhibited cardiomyocyte injury and NLRP3 inflammasome-mediated pyroptosis.
3. PNS inhibited cardiomyocyte pyroptosis by downregulating lncRNA-ANRIL.

**Supplementary Information:**

The online version contains supplementary material available at 10.1186/s13020-023-00756-2.

## Introduction

Cardiorenal syndrome (CRS) is a complex pathophysiological disease of heart and kidney, in which dysfunction of one usually induces injury of the other [1]. The association between chronic kidney disease (CKD) and the following chronic cardiovascular disease has been defined as CRS type 4 (CRS4), which is the progression of cardiac failure and cardiac complications in patients with CKD [2, 3]. CKD has been an important healthcare problem with global morbidity of approximately 13%, and the high morbidity of CRS4 has resulted in a huge burden on public health [4, 5]. Several treatments for cardiovascular and renal diseases have been proven significant in clinical. However, the treatment of CRS4 is still a major challenge, because some drugs showing efficiency in one organ failure may worsen the function of the other [1]. Therefore, it is essential to explore CRS4 in depth and find novel treatment strategies for it.

More and more studies have demonstrated that pyroptosis plays a crucial role in the progression of cardiovascular diseases [6]. Pyroptosis is a kind of pro-inflammatory cell death mediated by the Gasdermin family [7]. Activated NLRP3 inflammasome induces caspase-1 activation, which drives the maturation of IL-1β and then leads to pyroptosis [8]. Expression of NLRP3/caspase-1/IL-1β has been reported in patients with diabetic cardiomyopathy, myocardial infarction, and heart failure [9–11]. However, there are rare studies about the correlation between pyroptosis and CRS4.

*Panax notoginseng* is a traditional Chinese herb that has been used to promote blood circulation, dissolve stasis, and manage bleeding for hundreds of years [12]. *Panax notoginseng* saponins (PNS) is the major bioactive substance in *Panax notoginseng* [13]. Many studies have confirmed that PNS can combat cardiovascular diseases, because of its function of antithrombosis, antioxidation, anti-inflammation, and anti-hyperlipidemia [13, 14]. PNS has been reported to protect cardiomyocytes from endoplasmic reticulum stress, and inhibit cardiomyocyte apoptosis and oxidative stress-induced cardiac cell damage [14–16]. Wang et al. revealed that PNS ameliorated acute myocardial infarction and heart failure through autophagy [17]. Moreover, PNS also showed efficiency in kidney diseases, which can inhibit the development of inflammation and fibrosis in the kidney tissue [18]. A previous study demonstrated that PNS reduced cisplatin-induced acute renal injury by inhibiting mitochondrial apoptosis [19]. All of these results indicate that PNS exhibits therapeutic effects on heart and kidney diseases. However, the role of PNS in CRS4 has been rarely reported.

The expression of long non-coding RNA (lncRNA) was affected by PNS treatment in cardiomyocytes [20]. LncRNA is a class of RNAs that play crucial roles in cardiovascular diseases by regulating diverse physiological processes, such as cardiac myocyte apoptosis and autophagy, inflammation, and myocardial fibrosis [21]. LncRNA antisense non-coding RNA in the INK4 locus (ANRIL) is a crucial regulator involved in the pathogenesis of cardiovascular disorders [22]. Overexpression of ANRIL is reported to relate to poor prognosis of coronary heart disease [23]. In an animal model of acute myocardial infarction, ANRIL downregulation alleviated cardiomyocyte apoptosis and improved heart function [24]. ANRIL also mediated endothelial dysfunction in kidney injury [25]. ANRIL downregulation was reported to inhibit autophagy of cardiomyocytes in mice with uremia [26]. Previous experiments demonstrated that ANRIL could identify the risk of major harmful cardiovascular events in hemodialysis patients, which reveals that ANRIL participates in the progression of cardiovascular complications in CKD [27].

Based on the aforementioned research, we speculate that PNS inhibits pyroptosis in CRS4 by targeting ANRIL. In the present study, CRS4 model rats and hypoxia-induced cardiomyocytes were used to explore the function and mechanism of PNS in CRS4. This study aimed to provide original insights into the therapeutic value of PNS against CRS4.

## Methods

### High-performance liquid chromatography (HPLC) analysis

PNS samples (S27243) and standard solutions of notoginsenoside R1 (B21099) and ginsenoside (Rg1, B21057; Re, B21055; Rb1, B21050; Rd, B21054) were purchased from Yuanye Bio-Technology Co. Ltd. (Shanghai, China). A 25 mg sample of PNS was placed in a 10 mL volumetric flask. Approximately 8 mL of 70% methanol was added, followed by ultrasonic treatment (640 W, 40 kHz) for 30 min. Then diluted to the mark with 70% methanol and mixed thoroughly.

HPLC were performed on the Agilent HPLC 1260 series system (Agilent, CA, USA). Samples were separated using C_18_ reversed phase Eclipse XDB column (5 μm, 4.6 mm × 250 mm). A flow rate of 1.3 mL/min and sample injection volume of 10 μL were employed. The detection wavelength was set at 203 nm, and the column temperature was maintained at 25 °C. The mobile phase comprised acetonitrile (A) and water (B), with a linear gradient established as follows: 0–20 min for 20% A, 20–55 min for 20% A to 46% A, and 55–60 min for 46% A.

### Animal model

Animal experiments were approved by the Animal Ethics Committee of Yangzhou University (202209005). Sprague Dawley rats (male; 6-week-old, weighing 160 ± 20 g; n = 30) purchased from GemPharmatech (Jiangsu, China) were fed in a space at 23 ± 3 °C (12 h/12 h light/dark cycle, 44 ± 2% humidity) with access to water and food ad libitum. All rats were divided into five groups (n = 6): sham, model, model + 50 mg/kg d^−1^ PNS, model + 100 mg/kg d^−1^ PNS, and model + 200 mg/kg d^−1^ PNS groups. The CRS4 rat model was constructed as previously described [5]. Briefly, all rats were anesthetized intraperitoneally with 40 mg/kg ketamine and 5 mg/kg xylazine (Sigma-Aldrich, MO, USA) and fixed at a 37 °C electrical warming pad to keep body temperature. The artery of left kidney was momentarily occluded before the upper and lower poles of this kidney were ligated and removed. The left kidney remained with a third of it in this manner. Buprenorphine (0.03 mg/kg) was subcutaneously injected twice daily for 3 days to maintain postoperative analgesia. One week after, the right kidney was removed following the occlusion of renal pedicle. Sham rats received a similar procedure with exception that only renal envelopes were wiped off. PNS treatment rats were administered intragastrically with 50 mg/kg d^−1^, 100 mg/kg d^−1^, or 200 mg/kg d^−1^ of PNS, while rats in model group received saline solution.

Eight weeks after modeling, blood samples were collected via the tail vein for further experiments. The 24 h urine was collected using metabolic cages, and the urine protein was measured by an automatic chemistry analyzer (Thermo Fisher Scientific, MA, USA).

### Echocardiography

All rats were anesthetized by intraperitoneally with 50 mg/kg sodium pentobarbital and put on a 37 °C pad in a supine position. According to manufacturer’s instructions, the 2D M-mode echocardiography was recorded through the Vevo 2100 ultrasound machine (Visual Sonics, Canada) with a 35 MHz ultrasound probe. Left ventricular posterior wall thickness at diastole (LVPWd), left ventricular internal diameter systolic (LVIDs), left ventricular ejection fraction (LVEF), and left ventricular fractional shortening (LVFS) were measured thrice and averaged.

### Masson staining

All rats were sacrificed by intraperitoneally injecting with 4% sodium pentobarbital (200 mg/kg). After being excised and washed with phosphate-buffered saline (PBS; Beyotime, Shanghai, China), cardiac samples were fixed in 4% paraformaldehyde for 24 h, embedded in paraffin, and sliced into 4 μm sections. The slices were then stained with Weigert hematoxylin for 10 min and differentiated in 1% molybdenum phosphate acid. After being treated with decolorized blue aniline, slices were differentiated with 0.2% glacial acetic acid. Finally, the sections were dehydrated in absolute ethanol and rinsed in xylene. Light microscope (Olympus) was utilized to observe the slices.

### Cell culture and treatment

H9c2 cells were supplied by Stem Cell Bank, Chinese Academy of Science (Shanghai, China) and cultured in high-glucose (33.3 mM) Dulbecco’s modified Eagle’s medium (DMEM; Hyclone, UT, USA) with 10% fetal bovine serum and 1% penicillin–streptomycin at 37 °C with 5% CO_2_ and constant humidity.

To construct hypoxia-injured cells, H9c2 cells were incubated in an atmosphere of 94% N_2_, 5% CO_2_, and 1% O_2_ for 4 h. Hypoxia-injured cells were cultured in 100, 200, or 400 μg/mL PNS for a further 4 h after hypoxia injury. Part of the hypoxia-injured cells was treated with 400 μg/mL PNS and/or 50 μΜ VX-765 (an inhibitor of caspase-1) for 4 h, and cellular morphology was surveyed by electron microscope (Olympus, Tokyo, Japan).

### Cell transfection

Hypoxia-injured cells (2 × 10^5^ mL/well) were seeded into six-well plates. ANRIL-overexpression (oe-ANRIL) pcDNA3.1 plasmid or negative control (NC, empty plasmid) were transfected into H9c2 cells using Lipofectamine 2000 reagent (Invitrogen, CA, USA). After transfection for 18 h, cells were incubated in a fresh DMEM for 48 h. Subsequently, stably transfected cells were treated with 400 μg/mL PNS for 4 h.

### Cell counting kit-8 (CCK-8) assay

H9c2 cells (100 μL/well) were grown in 96-well plates at 37 °C with 5% CO_2_. Followed by 48 h-corresponding drug treatment, cells were treated with CCK-8 reagent (10 μL; Beyotime, China) for another 2 h. The optical density (OD) value at 450 nm was determined using microplate reader (Wuxi Hiwell Diatek, Jiangsu, China).

### Flow cytometry for cell apoptosis

The apoptosis was determined using Annexin V-EGFP/propidium iodide (PI) double staining kit (Beyotime). Briefly, cells were trypsin digested and centrifugated at 1500 rpm for 5 min. After being resuspended with 195 μL Annexin V-EGFP binding solution, cells were treated with 5 μL Annexin V-EGFP and 10 μL PI staining for 20 min in dark. The apoptosis rate was analyzed using the FACSCanto flow cytometer (BD Biosciences, NJ, USA).

### Immunofluorescence staining

H9c2 cells (5 × 10^5^ cells/well) were added into 12-well plates. After fixing in 3% paraformaldehyde for 10 min, cells were washed with PBS (Beyotime) and permeabilized by 1% Triton X-100 (Solarbio, Beijing, China) for 5 min. To determine cell pyroptosis, H9c2 cells were stained with the PI solution for 15 min before being stained with 4ʹ,6-diamidino-2-phenylindole (DAPI; Beyotime) for 5 min at room temperature in dark. Cells were observed under a fluorescence microscope (Olympus).

For detection of expression on gasdermin D (GSDMD-N) and caspase-1, H9c2 cells were further sealed with 3% BSA solution (Solarbio) for 30 min. Next, cells were incubated with primary antibodies anti-N terminal of GSDMD-N (DF13758; 1:100, Affinity, TX, USA) and anti-caspase-1 (ab286125; 1:100, Abcam, Cambridge, UK) at 4 °C overnight and treated with secondary antibody FITC goat anti-rabbit IgG (H + L) (AS011; 1:500, ABclonal Technology, MA, USA) for a further 30 min at room temperature. Nucleus was stained by DAPI (Beyotime). The images were captured under a confocal microscope (Zeiss Microscopy, Jena, Germany).

### Enzyme-linked immunosorbent assay (ELISA)

Corresponding ELISA kits were used to determine the levels of creatine kinase isoenzymes (CK-MB; ml092665; mlbio, Shanghai, China), lactate dehydrogenase (LDH; ml003416; mlbio), brain natriuretic peptide (BNP; ml003039; mlbio), blood urea nitrogen (BUN; ml092695; mlbio), plasma sulfate (IS; ab252894; Abcam), and serum creatinine (Scr; ml092663; mlbio). Cell suspension or cardiac serum was added into 50 μL standards in the testing sample wells, and blank wells were left empty. After treatment with 100 µL horseradish peroxidase-labeled enzyme conjugate working solution, cells or cardiac serum were sealed and incubated at 37 °C for 0.5 h. Subsequently, 50 µL substrates A and 50 µL B were added into each well at 37 °C for 15 min in dark, and 50 µL stop solution was then added to each well for 15 min treatment. OD values (CK-MB at 340 nm, BNP and LDH at 540 nm, Scr at 546 nm, BUN at 520 nm, and IS at 600 nm) were detected with a microplate reader (Wuxi Hiwell Diatek).

### Quantitative reverse transcription-polymerase chain reaction (RT-qPCR)

Total RNA of cardiac tissues or cells were extracted using Trizol reagent (Invitrogen) and reversely transcribed to cDNA with a Prime-Script reagent kit (Tiangen, Beijing, China). SYBR Green kit was used to detect relative mRNA levels on a 7500 Fast RT-PCR System (Biosystem, Singapore). The thermal cycling parameters were: 95 °C for 3 min; 45 cycles of 95 °C for 12 s and 62 °C for 40 s. GAPDH was utilized as an internal reference. Gene expression was determined through the 2^−ΔΔCT^ method. Primer sequences were presented in Table 1.

**Table 1:** Primer sequences in RT-qPCR

Genes	Primer sequences (5ʹ-3ʹ)
Rat COL-I forward	TACCCTGGCAACATTGGTCC
Rat COL-I reverse	CTGAGAAGCACGGTTGGCTA
Rat COL-III forward	GCCTTCTACACCTGCTCCT
Rat COL-III reverse	CCACTCCAGACTTGACATCATAT
Rat TGF-β forward	CATGAACGGGCAGTGCAAAA
Rat TGF-β reverse	TATCCTCGCAGTGGTCTCCA
Rat α-SMA forward	AGGTAACGAGTCAGAGCTTTGGC
Rat α-SMA reverse	CTCTCTGTCCACCTTCCAGCAG
Rat ANRIL forward	CAAGCCACGTTGGAAGATGC
Rat ANRIL reverse	AGAGTGTGTAGCAGCTGACG
Rat GAPDH forward	GCGAGATCCCGCTAACATCA
Rat GAPDH reverse	CTCGTGGTTCACACCCATCA

### Western blotting

Total proteins were extracted from cardiac tissues and H9c2 cells by radioimmunoprecipitation assay lysis buffer (Beyotime) and measured through a bicinchoninic acid protein assay kit (Beyotime). Protein samples (20 μg) were isolated by 10% SDS-PAGE (Beyotime) and transferred into polyvinylidene fluoride (PVDF; Beyotime) membranes. PVDF membranes were then treated with 5% skimmed milk/TBST for 1 h. Subsequently, the membranes were treated with primary antibodies overnight at 4 °C and secondary antibody for another 2 h at ambient temperature. The primary antibodies were as follows: anti-interleukin (IL)-1β (ab254360; 1:1000, Abcam), anti-nucleotide-binding oligomerization domain (NLRP3; ab263899; 1:1000, Abcam), anti-GSDMD-N (DF13758; 1:1000, Affinity), anti-caspase-1 (ab286125; 1 μg/mL, Abcam), anti-ASC (ab180799; 1:1000, Abcam), anti-transforming growth factor (TGF-β1; ab215715; 1:1000, Abcam), and anti-GAPDH (ab181602; 1:1000, Abcam). GAPDH was used as an internal reference. Finally, protein bands were visualized with an electrochemical luminescence on a Tanon 5200 machine (Tanon, Shanghai, China). Protein band intensities were detected with the Image J software (NIH, MD, USA).

### Statistical analysis

Each assay was repeated at least in triplicate. Data are expressed as mean ± standard deviation. Statistical analysis was performed using GraphPad Prism software 8.0. A one-way analysis of variance followed by Tukey’s test was utilized for comparisons of multiple groups. *p* < 0.05 was regarded as statistically significant.

## Results

### Analysis of PNS contents

Results of HPLC were shown in Additional file 1: Figure S1. Notoginsenoside R1 and ginsenoside (Rg1, Re, Rb1, Rd) were identified in the PNS samples, with concentrations of 4.51446 ng/μL, 1.63831 ng/μL, 2.05892 ng/μL, 1.86199 ng/μL, and 3.97754 ng/μL, respectively.

### PNS alleviates cardiac dysfunction, cardiac fibrosis, and pyroptosis in CRS4 model rats

**Fig. 1:**
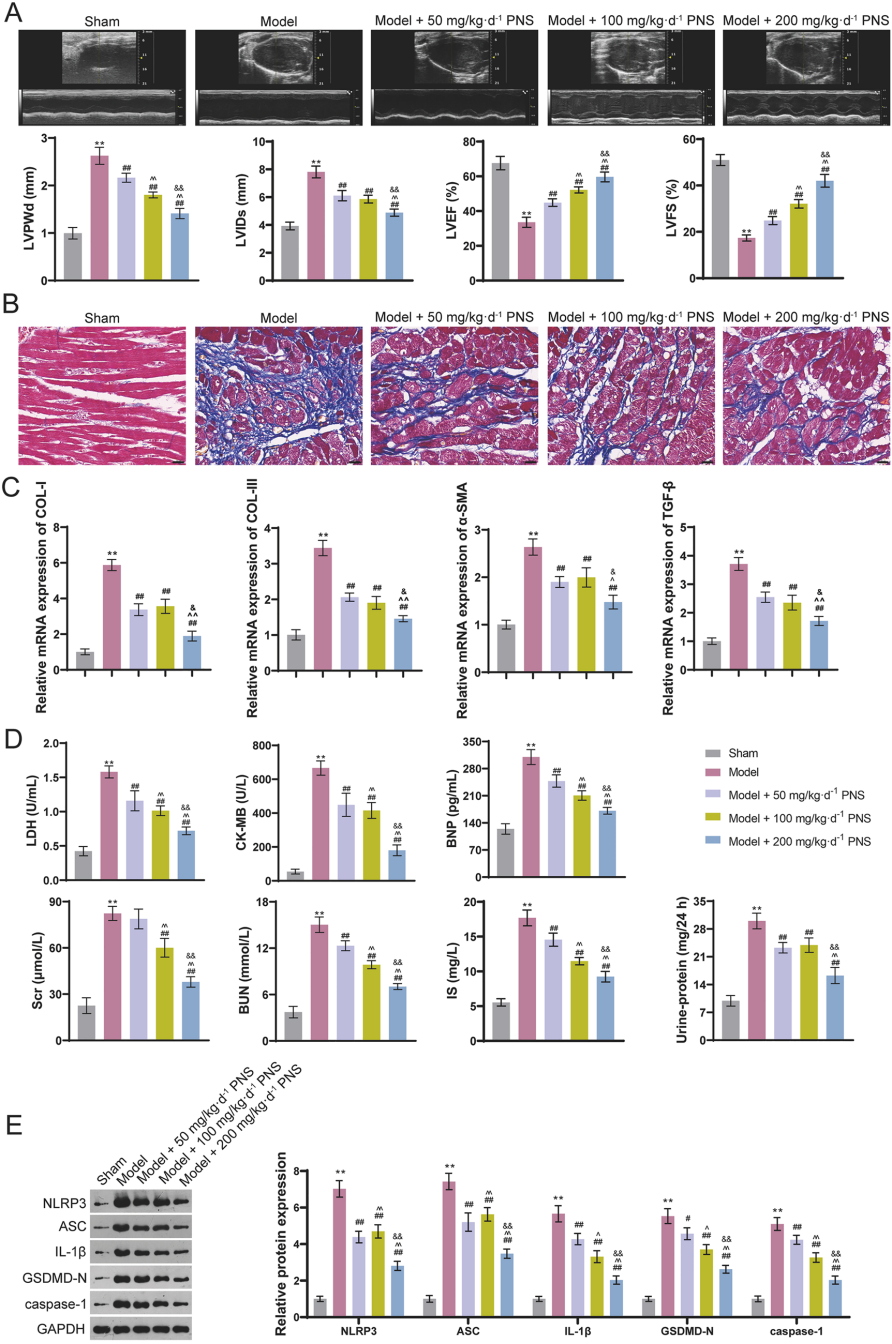
PNS alleviates cardiac dysfunction, cardiac fibrosis, and pyroptosis in CRS4 model rats. **A** Echocardiography determines LVPWd, LVIDs, LVEF, and LVFS. **B** Masson staining for cardiac tissues; scale bar = 20 μm. **C** RT-qPCR was used to measure the relative mRNA expression of fibrosis-associated genes (COL-I, COL-III, TGF-β, and α-SMA) in cardiac tissues. **D** Levels of LDH, CK-MB, BNP, Scr, IS and BUN in serum was determined by ELISA, and 24 h urine protein was determined by an automatic chemistry analyzer. **E** Western blotting was used to determine the protein expression levels of NLRP3 inflammasome-related proteins (NLRP3, ASC, IL-1β) and pyroptosis-related proteins (GSDMD-N, caspase-1) in cardiac tissues. Male Sprague Dawley rats received 5/6 subtotal nephrectomy to construct a CRS4 rat model; model rats were administered intragastrically with corresponding doses of PNS (50 mg/kg d^−1^, 100 mg/kg d^−1^, and 200 mg/kg d^−1^). **p < 0.01 vs. sham group; ^#^*p* < 0.05, ^##^*p* < 0.01 vs. model group; ^^^*p* < 0.05, ^^^^*p* < 0.01 vs. model + 50 mg/kg d^−1^ PNS group; ^&^*p* < 0.05, ^&&^*p* < 0.01 vs. model + 100 mg/kg d^−1^ PNS group. PNS: *Panax notoginseng* saponins; CRS4: Cardiorenal syndrome type 4; LVPWd: left ventricular posterior wall thickness at diastole; LVIDs: left ventricular internal diameter systolic; LVEF: left ventricular ejection fraction; LVFS: left ventricular fractional shortening; LDH: Lactate dehydrogenase; CK-MB: Creatine kinase isoenzymes; BNP: brain natriuretic peptide; BUN: blood urea nitrogen; IS: plasma sulfate; Scr: serum creatinine; COL: collagen; TGF: transforming growth factor; IL: interleukin; GSDMD-N: N terminal of gasdermin D

Firstly, we evaluated effects of PNS on cardiac function in CRS4 rats. Echocardiography showed that the cardiac function biomarkers of LVPWd and LVIDs were increased, whereas LVEF and LVFS were reduced in model rats compared with that in sham rats (*p* < 0.01; Fig. 1A). Masson staining was performed to observe fibrosis of the heart, and it showed obvious cardiac fibrosis in model rats (Fig. 1B). Furthermore, the mRNA expression of fibrosis-associated genes (COL-I, COL-III, α-SMA, and TGF-β) was upregulated in model rats compared to that in sham rats (*p* < 0.01; Fig. 1C). The contents of cardiac injury biomarkers (CK-MB and LDH) and heart failure biomarker (BNP) were increased in the model rats compared to those in sham rats (*p* < 0.01; Fig. 1D). Besides, the levels of renal function biomarkers (BUN, IS, Scr, and 24 h urine protein) were all increased in the model rats (*p* < 0.01; Fig. 1D). Results revealed that PNS enhanced cardiac function in a dose-dependent manner, as shown by evidence of decreased LVPWd and LVIDs and increased LVEF and LVFS in model rats (*p* < 0.01; Fig. 1A). PNS also ameliorated cardiac fibrosis (Fig. 1B) and downregulated expression of COL-I, COL-III, α-SMA, and TGF-β (*p* < 0.01; Fig. 1C). Additionally, PNS reduced the contents of CK-MB, LDH, BNP, BUN, IS, Scr, and 24 h urine protein (*p* < 0.01; Fig. 1D). Noticeably, compared with 100 mg/kg d^−1^ PNS, 200 mg/kg d^−1^ PNS showed a better inhibitory effect on the levels of fibrosis-related genes and the above biomarkers (*p* < 0.05; Fig. 1C and D).

Pyroptosis is a highly regulated cell death process, whose inhibition has been confirmed to be cardioprotective in cardiovascular diseases [28]. To explore whether PNS affects pyroptosis in CRS4, expression levels of pyroptosis-related proteins were measured in model rats. The expression levels of NLRP3 inflammasome-related proteins (NLRP3, ASC, and IL-1β) and pyroptosis-related proteins (GSDMD-N and caspase-1) were all elevated in model rats compared with those in sham rats (*p* < 0.01; Fig. 1E). However, PNS inhibited these protein expression levels (*p* < 0.01; Fig. 1E), and 200 mg/kg d^−1^ PNS showed a better efficiency than 100 mg/kg d^−1^ PNS (*p* < 0.01; Fig. 1E).

### PNS reduces hypoxia-induced injury, apoptosis, and pyroptosis of cardiomyocytes

**Fig. 2:**
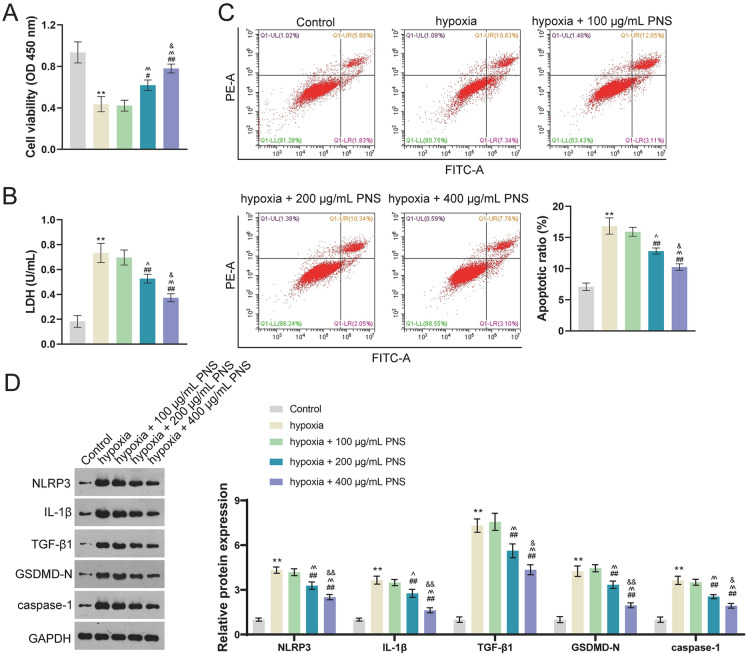
PNS reduces hypoxia-induced injury, apoptosis, and pyroptosis in cardiomyocytes. **A** Cell viability was determined by CCK-8. **B** LDH was measured by ELISA. **C** Cell apoptosis was determined by flow cytometry assay. **D** Western blotting was used to determine protein expression levels of NLRP3, IL-1β, TGF-β1, GSDMD-N, and caspase-1. H9c2 cells were incubated in an atmosphere of 94% N_2_, 5% CO_2_, and 1% O_2_ for 4 h and treated with corresponding doses of PNS (100 μg/mL, 200 μg/mL, and 400 μg/mL). **p < 0.01 vs. control group; ^#^*p* < 0.05, ^##^*p* < 0.01 vs. hypoxia group; ^^^*p* < 0.05, ^^^^*p* < 0.01 vs. hypoxia + 100 μg/mL PNS group; ^&^*p* < 0.05, ^&&^*p* < 0.01 vs. hypoxia + 200 μg/mL PNS group. PNS: *Panax notoginseng* saponins; CCK-8: cell counting kit-8; LDH: Lactate dehydrogenase; TGF: transforming growth factor; IL: interleukin; GSDMD-N: N terminal of gasdermin D

Subsequently, we explored the effect of PNS on injured cardiomyocytes. H9c2 cells were damaged in the hypoxia atmosphere. CCK-8 revealed that cell viability was dramatically reduced in hypoxia group compared with control group (*p* < 0.01; Fig. 2A). In addition, content of cardiac injury biomarker (LDH) was increased in hypoxia group compared to control group (*p* < 0.01; Fig. 2B). In comparison with control group, cell apoptosis was enhanced in hypoxia group (*p* < 0.01; Fig. 2C). The protein expression levels of NLRP3, IL-1β, TGF-β1, GSDMD-N, and caspase-1 were all elevated in hypoxia group (*p* < 0.01; Fig. 2D). After PNS treatment, cell viability was enhanced and LDH content in injured H9c2 cells was decreased (*p* < 0.05; Fig. 2A and B). Besides, 200 μg/mL and 400 μg/mL PNS also suppressed apoptosis of injured H9c2 cells (*p* < 0.01; Fig. 2C). It is noticed that 400 μg/mL PNS showed a higher efficiency on cell viability than 200 μg/mL PNS (*p* < 0.05; Fig. 2A–C). PNS also reduced expression levels of NLRP3, IL-1β, TGF-β1, GSDMD-N, and caspase-1 in injured H9c2 cells. Besides, 400 μg/mL PNS showed better inhibitory effect on cell pyroptosis than 200 μg/mL PNS (*p* < 0.05; Fig. 2D).

### PNS inhibits NLRP3 inflammasome-mediated pyroptosis

**Fig. 3:**
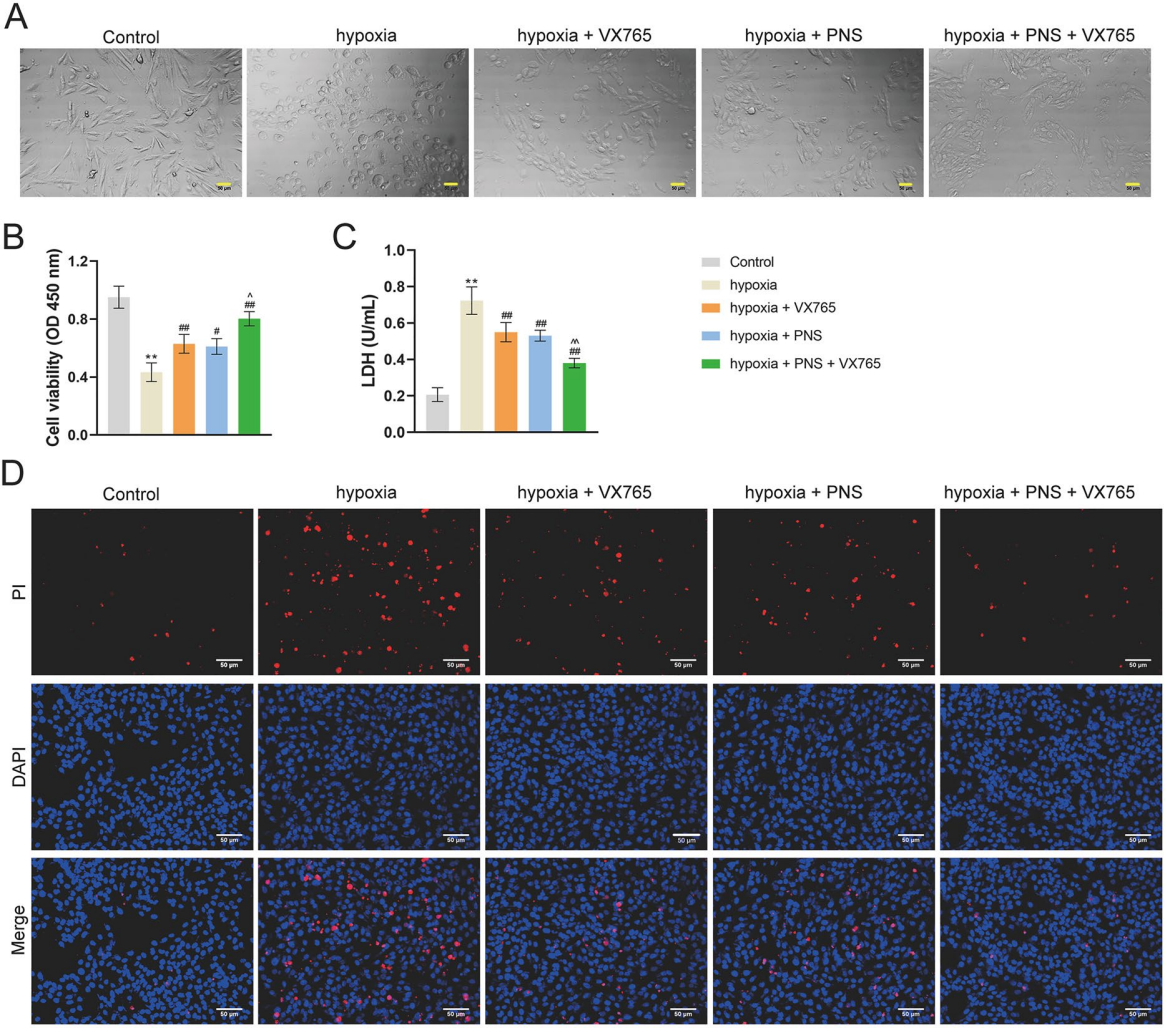
PNS inhibits cardiomyocyte pyroptosis. **A** H9c2 cellular morphology was observed by electron microscope; scale bar = 50 μm. **B** Cell viability was determined by CCK-8. **C** LDH was measured by ELISA. **D** Cell pyroptosis was detected by PI staining; scale bar = 50 μm. H9c2 cells were incubated in an atmosphere of 94% N_2_, 5% CO_2_, and 1% O_2_ for 4 h and treated with VX765 (a specific pyroptosis inhibitor) and/or 400 μg/mL PNS. **p < 0.01 vs. control group; ^#^*p* < 0.05, ^##^*p* < 0.01 vs. hypoxia group; ^^^*p* < 0.05, ^^^^*p* < 0.01 vs. hypoxia + PNS group. PNS: *Panax notoginseng* saponins; CCK-8: cell counting kit-8; LDH: Lactate dehydrogenase; PI: propidium iodide

Pyroptosis has an important role in cardiac injury [29]. In the above experiments, results indicated that PNS reduced the pyroptosis-related protein expression in vivo and in vitro. Therefore, we then surveyed the relationship between PNS and pyroptosis in injured H9c2 cells. Cell swelling is a typical morphological change of pyroptosis. As shown in Fig. 3A, cells in control group are spindle-shaped, but those in the hypoxia group are rounded, swelled, and even broken. However, PNS and/or VX765 (a specific pyroptosis inhibitor) reduced the degree of cell distension. Additionally, PNS and/or VX765 treatment enhanced cell viability while decreasing LDH level in injured cells (*p* < 0.05; Fig. 3B and C). Co-treatment of PNS and VX765 showed better therapeutic efficiency than PNS or VX765 single treatment (*p* < 0.05; Fig. 3B and C). Cell pyroptosis was promoted in hypoxia group compared to control group, whereas PNS and/or VX765 treatment suppressed it (Fig. 3D). Besides, PNS and VN765 co-treatment exhibited a better inhibitory effect on cell pyroptosis (Fig. 3D).

**Fig. 4:**
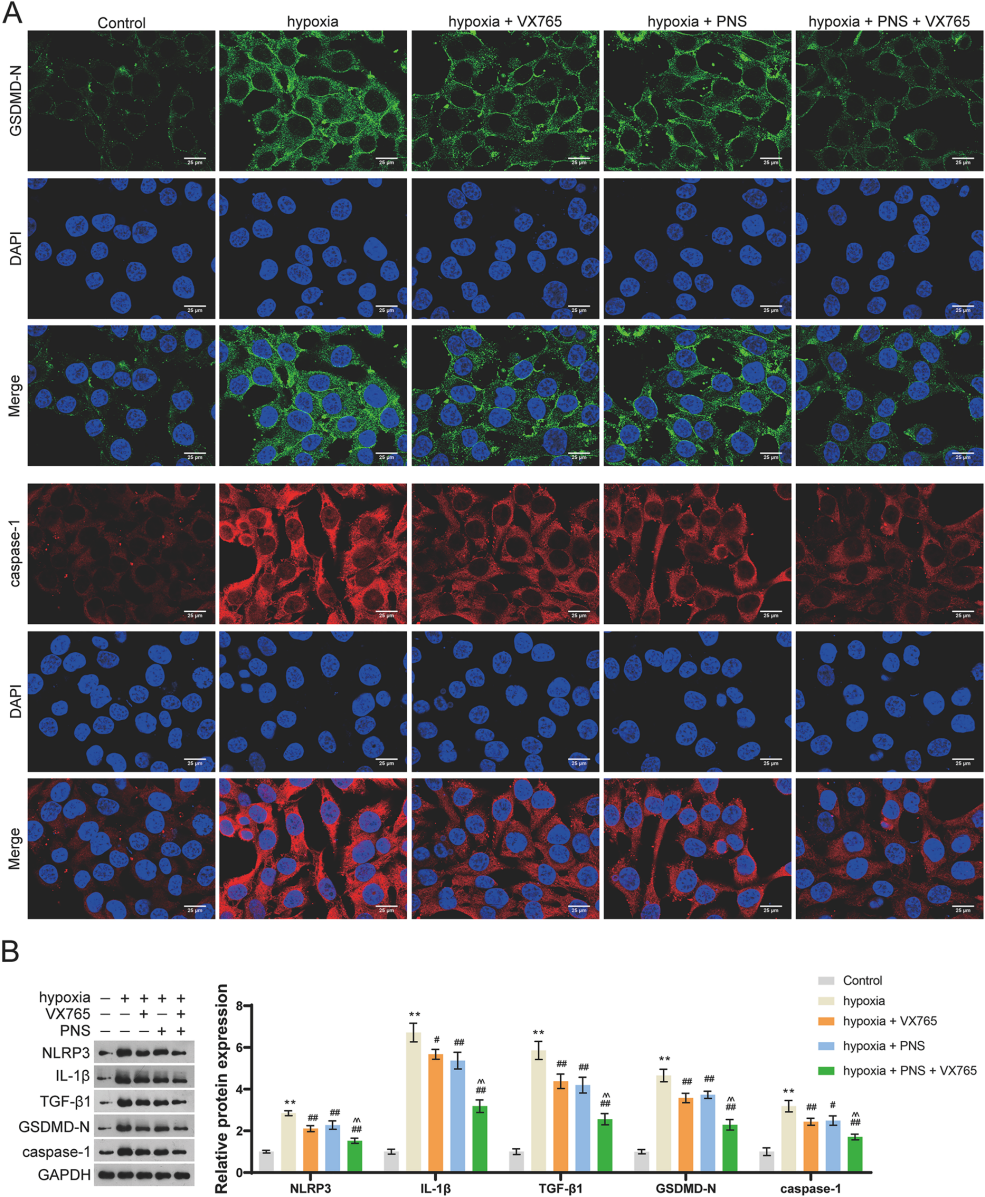
PNS inhibits NLRP3 inflammasome-mediated cardiomyocyte pyroptosis. **A** Protein expression levels of GSDMD-N and caspase-1 were measured by immunofluorescence staining; scale bar = 25 μm. **B** Western blotting was used to determine protein expression levels of NLRP3, IL-1β, TGF-β1, GSDMD-N, and caspase-1. H9c2 cells were incubated in an atmosphere of 94% N_2_, 5% CO_2_, and 1% O_2_ for 4 h and treated with VX765 and/or 400 μg/mL PNS. **p < 0.01 vs. control group; ^#^*p* < 0.05, ^##^*p* < 0.01 vs. hypoxia group; ^^^^*p* < 0.01 vs. hypoxia + PNS group. PNS: *Panax notoginseng* saponins; TGF: transforming growth factor; IL: interleukin; GSDMD-N: N terminal of gasdermin D

Immunofluorescent staining also revealed that levels of GSDMD-N and caspase-1 were elevated in hypoxia group, which was reduced by PNS and/or VX765 treatment (Fig. 4A). Moreover, protein expression levels of NLRP3, IL-1β, TGF-β1, GSDMD-N, and caspase-1 were all retarded by PNS and/or VX765 treatment (*p* < 0.05; Fig. 4B). It is noticed that co-treatment of PNS and VX765 showed greater inhibitory effects on levels of these proteins (*p* < 0.05; Fig. 4A and B).

### PNS inhibits cardiomyocyte pyroptosis by downregulating lncRNA-ANRIL

**Fig. 5:**
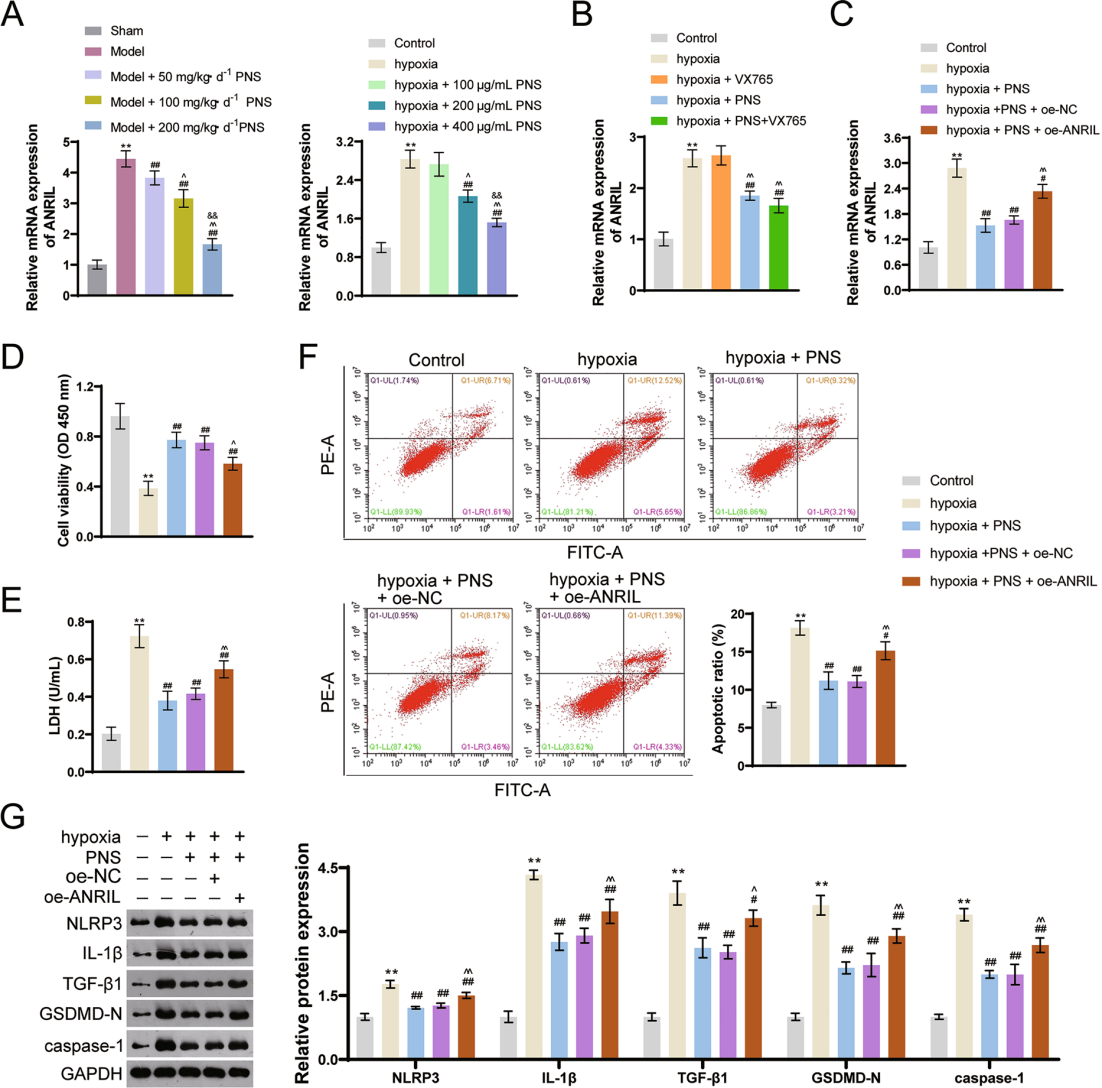
PNS inhibits cardiomyocyte pyroptosis by downregulating lncRNA-ANRIL. **A** RT-qPCR was used to measure the ANRIL mRNA expression in model rats and injured cells. Male Sprague Dawley rats received 5/6 subtotal nephrectomy to construct a CRS4 rat model; model rats were administered intragastrically with corresponding doses of PNS (50 mg/kg d^−1^, 100 mg/kg d^−1^, and 200 mg/kg d^−1^); **p < 0.01 vs. sham group; ^##^*p* < 0.01 vs. model group; ^^^*p* < 0.05, ^^^^*p* < 0.01 vs. model + 50 mg/kg d^−1^ PNS group; ^&&^*p* < 0.01 vs. model + 100 mg/kg d^−1^ PNS group. H9c2 cells were incubated in an atmosphere of 94% N_2_, 5% CO_2_, and 1% O_2_ for 4 h and treated with corresponding doses of PNS (100 μg/mL, 200 μg/mL, and 400 μg/mL); **p < 0.01 vs. control group; ^##^*p* < 0.01 vs. hypoxia group; ^^^*p* < 0.05, ^^^^*p* < 0.01 vs. hypoxia + 100 μg/mL PNS group; ^&&^*p* < 0.01 vs. hypoxia + 200 μg/mL PNS group. **B** RT-qPCR was used to measure the ANRIL mRNA expression in H9c2 cells. H9c2 cells were incubated in an atmosphere of 94% N_2_, 5% CO_2_, and 1% O_2_ for 4 h and treated with VX765 and/or 400 μg/mL PNS. **p < 0.01 vs. control group; ^##^*p* < 0.01 vs. hypoxia group; ^^^^*p* < 0.01 vs. hypoxia + PNS group. **C** RT-qPCR measured the ANRIL mRNA expression in H9c2 cells. **D** Cell viability was determined by CCK-8. **E** LDH was measured by ELISA. **F** Cell apoptosis was determined by flow cytometry assay. **G** Western blotting was used to determine protein expression levels of NLRP3, IL-1β, TGF-β1, GSDMD-N, and caspase-1. **C**–**G** H9c2 cells were incubated in an atmosphere of 94% N_2_, 5% CO_2_, and 1% O_2_ for 4 h, treated with 400 μg/mL PNS, and transfected with oe-NC/oe-ANRIL. **p < 0.01 vs. control group; ^#^*p* < 0.05, ^##^*p* < 0.01 vs. hypoxia group; ^^^*p* < 0.05, ^^^^*p* < 0.01 vs. hypoxia + PNS + oe-NC group. PNS: *Panax notoginseng* saponins; LncRNA: long non-coding RNA; ANRIL: antisense non-coding RNA in the INK4 locus (ANRIL); CCK-8: cell counting kit-8; LDH: Lactate dehydrogenase; TGF: transforming growth factor; IL: interleukin; GSDMD-N: N terminal of gasdermin D

ANRIL is an important regulator related to the pathogenesis of cardiovascular diseases. To investigate the underlying mechanism of PNS in CRS4, ANRIL expression was detected both in vivo and in vitro. Results showed that ANRIL was upregulated in model rats and injured cardiomyocytes (*p* < 0.01; Fig. 5A). PNS inhibited expression of ANRIL, but VX765 showed no significance in its expression (Fig. 5A, B). Subsequently, oe-ANRIL was transfected into PNS-treated injured H9c2 cells. As shown in Fig. 5C, ANRIL expression was upregulated in oe-ANRIL cells (*p* < 0.01), which indicated a successful transfection of oe-ANRIL. ANRIL overexpression reversed the effects of PNS by decreasing cell viability, increasing LDH level, and promoting cell apoptosis (*p* < 0.05; Fig. 5D–F). Furthermore, levels of NLRP3, IL-1β, TGF-β1, GSDMD-N, and caspase-1 were all reversed by ANRIL upregulation (*p* < 0.05; Fig. 5G).

## Discussion

CRS4 has arisen growing attention because of its high morbidity and mortality. In the study, we found that PNS alleviated cardiac fibrosis and improved cardiac function in a CRS4 rat model. Furthermore, the cell experiments verified that CRS4 promoted cardiomyocyte viability, and inhibited apoptosis and NLRP3 inflammasome-induced pyroptosis. Moreover, lncRNA-ANRIL overexpression was observed in cardiac tissues of CRS4 model rats and injured cardiomyocytes, which was reversed by PNS. Our data demonstrated that PNS inhibited apoptosis and pyroptosis by downregulating ANRIL in hypoxia-induced injured cardiomyocytes.

CRS4 is characterized by left ventricular hypertrophy (LVH), systolic dysfunction, later interstitial fibrosis, etc. [30]. In our study, obvious LVH was observed with evidence of increased LVPWd and LVIDs and decreased LVEF and LVFS in CRS4 rats. Levels of LDH, CK-MB, and BNP have been clinically utilized to evaluate heart injury [31], and increased levels of these biomarkers were observed in model rats as well as increased LDH in cardiomyocytes. In addition, Masson staining showed severe fibrosis in the cardiac tissues of model rats, and expression of fibrosis-related proteins (COL-I/III and α-SMA) was also upregulated in CRS4 rats. These phenomena revealed serious cardiac dysfunction in CRS4 rats. However, upon PNS treatment, cardiac function, fibrosis, and apoptosis were ameliorated, and levels of LDH, CK-MB, and BNP were also reduced. Levels of BUN, Scr, IS, and 24 h-urine protein are biomarkers of renal function [32]. In our study, their levels were increased in CRS4 rats but were reduced by PNS. These results demonstrate that PNS can protect against cardiac and renal injury in CRS4.

Pyroptosis is a recently identified programmed cell death that depends on inflammasomes and caspases [33]. In recent years, pyroptosis has been reported to play a critical role in various cardiovascular diseases [28]. For example, GSDMD-mediated pyroptosis promotes myocardial ischemia/reperfusion injury [34]; inflammasome-mediated pyroptosis is the main player in development of diabetic cardiomyopathy [35]. Herein, we explored the role of pyroptosis in CRS4. We observed that hypoxia-induced injured H9c2 cells were swelling, which is a typical cellular feature of pyroptosis. Besides, PI staining also showed more pyroptosis cells in hypoxia-induced injured group compared with control group. These indicate that pyroptosis participates in cardiomyocyte injury. Studies have validated that pyroptosis is driven by many receptors represented by the NLRP3, which is characterized by the release of caspase-1; caspase-1 is a canonical executor of pyroptosis by releasing GSDMD-N and IL-1β [36]. In the present study, upregulated expression of NLRP3, IL-1β, caspase-1, and GSDMD-N was observed in cardiac tissues of CRS4 model rats and injured cardiomyocytes. After PNS treatment, cell swelling and pyroptosis were reduced, which indicates that PNS suppresses pyroptosis in injured cardiomyocytes. A previous study reported that PNS downregulated expression of NLRP3 inflammasome pathway-related proteins in cerebral ischemia/reperfusion [37]. In our study, PNS also reduced expression of NLRP3, IL-1β, caspase-1, and GSDMD-N in model rats and injured H9c2 cells. Besides, we found that VX765, a specific inhibitor of caspase-1, enhanced the effect of PNS in injured H9c2 cells. These results reveal that PNS inhibits NLRP3 inflammasome-mediated pyroptosis in CRS4.

LncRNA extensively participates in important physiological processes such as metabolism and is associated with the progression of cancers, cardiovascular diseases and other diseases [38]. Studies have investigated function of lncRNA-ANRIL in cardiovascular diseases. ANRIL expression has been confirmed to predict coronary heart disease and coronary artery disease in the clinic [23, 39]. Yang et al. revealed that ANRIL knockdown relieves cardiomyocyte apoptosis in acute myocardial infarction by regulating IL-33/ST2 [24]. In our study, ANRIL was upregulated in CRS4 model rats and hypoxia-induced injured H9c2 cells. However, PNS reduced its expression in model rats and injured cardiomyocytes, which indicates that PNS may exert its therapeutic efficiency in CRS4 by targeting ANRIL. Our further experiments confirmed that ANRIL overexpression reversed the stimulative effect of PNS on cell viability in injured cardiomyocytes. These validate that PNS alleviates CRS4 by downregulating ANRIL. LncRNA has been confirmed to show a tight correlation with NLRP3 inflammasome-mediated pyroptosis in cardiovascular diseases [40]. For example, lncRNA-MALAT1 promotes H9c2 cardiomyocyte pyroptosis in diabetic cardiomyopathy [41]; lncRNA-PVT1 knockdown ameliorates pyroptosis of cardiomyocytes in myocardial ischemia/reperfusion [42]. In this study, ANRIL overexpression reversed inhibitory effect of PNS on the expression of NLRP3, IL-1β, caspase-1, and GSDMD-N in injured H9c2 cells. All of these results reveal that PNS inhibited NLRP3 inflammasome-mediated pyroptosis by downregulating ANRIL in injured cardiomyocytes.

There are some limitations in this study. First, the connection between ANRIL and pyroptosis in CRS4 should be explored in vivo. Second, the specific regulating mechanism between PNS and ANRIL was not fully explored, and the downstream targets of the ANRIL effect on pyroptosis in CRS4 need to be further explored. Further studies are also essential to investigate the pharmacologic effect of PNS on CRS4 in clinical.

## Conclusion

In conclusion, PNS improved cardiac function, decreased cardiac fibrosis, and inhibited pyroptosis in CRS4 rats. In vitro experiments revealed that PNS exhibited inhibitory effects on cardiomyocyte injury and NLRP3 inflammasome-mediated pyroptosis. Additionally, PNS reduced the expression of lncRNA-ANRIL, and our results indicate that PNS suppressed cardiomyocyte pyroptosis by downregulating lncRNA-ANRIL in CRS4. This study provided evidence of cardioprotective effects of PNS and its underlying mechanism involved in ANRIL in CRS4.

## Supplementary Information

**Additional file 1: Figure S1.** HPLC chromatogram profiles. Peak 1: Notoginseng R1; P2: Ginsenoside Rg1; P3: Ginsenoside Re; P4: Ginsenoside Rb1; P5: Ginsenoside Rd.

## Data Availability

The datasets used and analysed during the current study are available from the corresponding author on reasonable request.
