# Supplementary figures and images for: Panax notoginseng saponins inhibits NLRP3 inflammasome-mediated pyroptosis by downregulating lncRNA-ANRIL in cardiorenal syndrome type 4
Source: Chin Med. 2023 May 8;18:50. doi: 10.1186/s13020-023-00756-2 (PMC10165771; doi:10.1186/s13020-023-00756-2)

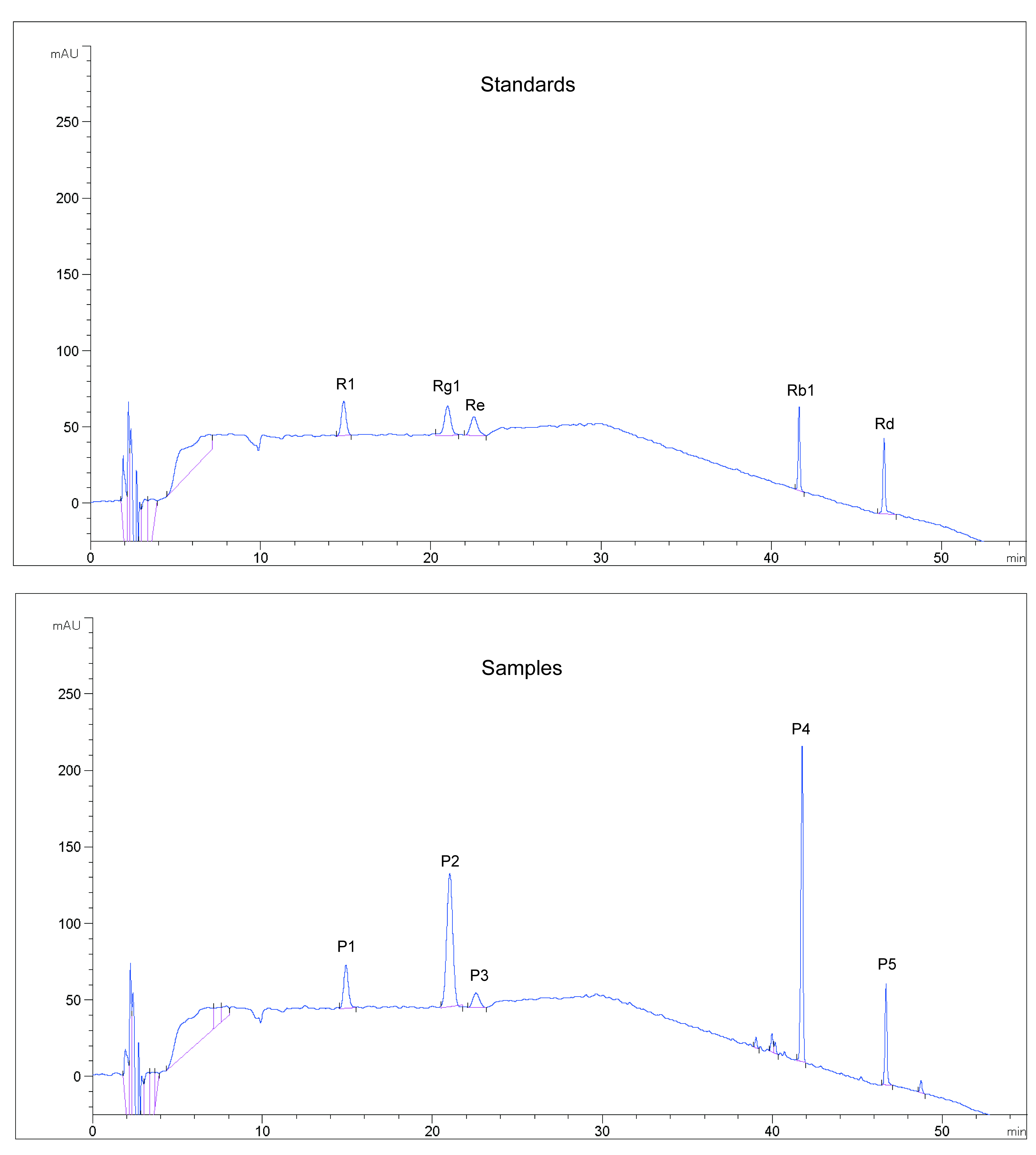

Supplement: Supplementary file 1 — Additional file 1: Figure S1. HPLC chromatogram profiles. Peak 1: Notoginseng R1; P2: Ginsenoside Rg1; P3: Ginsenoside Re; P4: Ginsenoside Rb1; P5: Ginsenoside Rd. [file 13020_2023_756_MOESM1_ESM.tif]
